# The Mediating Role of Self‐Control and Shame Between Sensitivity to Punishment and Intimacy

**DOI:** 10.1002/brb3.71543

**Published:** 2026-06-08

**Authors:** Zahra Mirzazade, Gholamreza Dehshiri, Shaghayegh Zahraie

**Affiliations:** ^1^ Department of Clinical Psychology Shiraz University Shiraz Iran; ^2^ Department of Psychology, Faculty of Education and Psychology Alzahra University Tehran Iran

**Keywords:** behavioral inhibition system, intimacy, reinforcement sensitivity theory, self‐control, sensitivity to punishment, shame

## Abstract

**Objectives:**

Interpersonal relationships are among the important aspects of life. Understanding the factors that might affect relationships can improve psychological health. This study aimed to examine the associations between sensitivity to punishment (SP) and intimacy and the mediating role of shame and self‐control.

**Methods:**

The population for this study included married people from Kerman, Iran. A total of 214 individuals completed the sensitivity to punishment and sensitivity to reward questionnaire revised and clarified (SPSRQ‐RC), experience of shame scale (ESS), Tangney self‐control scale short form, and personal assessment of intimate relationships questionnaire (PAIR).

**Results:**

The results were analyzed through Pearson correlation and a parallel mediation model. This study demonstrated that SP had a negative, significant association with intimacy. Shame and self‐control could significantly mediate this relationship.

**Conclusion:**

It is concluded that shame and self‐control can mediate the relationship between SP and intimacy. Learning how emotions and cognitive functions work in an individual's life might help improve intimacy.

## Graphical abstract

This study investigated the relationship between sensitivity to punishment, intimacy, and the mediating roles of shame and self‐control in married couples from Kerman, Iran. Results demonstrated a negative association between sensitivity to punishment and intimacy. Additionally, it was found that shame and self‐control can mediate this relationship. Therefore, the findings suggest that understanding how emotions and cognitive functions influence individuals’ lives can contribute to improving intimacy in relationships.

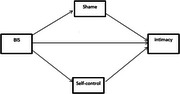

## 1 Introduction

Intimate relationships can enhance quality of life and reduce the risk of psychological disorders (Mikulincer and Shaver 2010). Research has revealed the importance of relationships and intimacy for quality of life and psychological health (Adams et al. 2023). Intimacy implies the ability to have close relationships and the ability to preserve them (Obert 2016). Although relational factors should be considered, personality factors may play important roles because of the consistent nature of the character (Mitchell et al. 2008). Greater knowledge of personality leads to a better understanding of needs in close relationships and communicate more effective (Yoo et al. 2014; Shahzadi and Walker 2019).

Gray's theory of personality (RST) explains how brain systems respond to different kinds of stimuli: Behavioral activation system (BAS) responds to rewarding stimuli, behavioral inhibition system (BIS) responds to conditional punishing stimuli, and fight–flight–freeze system (FFS) responds to unconditional and new punishing stimuli. According to this theory, individuals have exclusive sensitivity in each system that generates various personalities (Gray 1970, 1987). Therefore, individuals might experience the same emotions but cope with them differently.

In Gray's theory, the possibility of a certain behavior relies on the sensitivity of these brain systems. In other words, the more sensitive the BIS is, the greater the sensitivity to punishment (SP) (Izadpanah 2017). Consequently, vulnerability to anxiety, emotion dysregulation, and loss of self‐esteem are more probable (Hundt et al. 2013; Erdle and Rushton 2010). Individuals with high SP might use self‐attacks for everything, when bad situations and external limitations exist and then experience worry and rumination that cause more anxiety (Guimón et al. 2007; Corr 2008). In close relationships, they might interpret everything due to fear of being rejected. They probably avoid starting an intimate relationship. If they have been rejected or left before, negative emotions, such as shame, are experienced, and consequently, they avoid intimacy and feel lonely (Clark et al. 2015; Giovazolias and Paschalidi 2022).

According to operant interaction theory (Stuart 1969), relationships are dependent on reinforcing or destructive stimuli. This theory assumes that a couple's communication pattern results in good or bad signals that individuals can interpret according to their perceptions. Hence, sensitivity to stimuli can be an important factor in close relationships (Meyer et al. 2005).

High SP is associated with shame experience (Muris et al. 2015). Emotions, including shame, aim to improve decision‐making in social behaviors, and arousing them can illuminate the way for individuals (Lindsay‐Hartz 1984); however, when emotional arousal is severe, destructive behaviors may occur. Likewise, shame is an emotion with adaptive purposes; however, it can have harmful consequences when it exceeds normal levels. Experiencing this level of shame probably creates thoughts, such as having a defect, being worthless, and being inadequate (Tangney 1995). Therefore, these thoughts and feelings influence the individual's behaviors, including those that occur in intimate relationships (Lutwak et al. 2003).

Shame was initially understood as a self‐conscious emotion alongside feelings of guilt, embarrassment, pride, and so forth (Tangney 1995). However, recent research has shown that shame is not just an emotion but also extends to beliefs, behaviors, and self‐concept (DeYoung 2015). As a result, shame is a more painful and generalized experience (Schore and Schore 2008). Research suggests that although these emotions may co‐occur, they have distinct cognitive and motivational underpinnings (Gilbert et al. 2010; Lewis 2014). For example, shame tends to lead to withdrawal and avoidance, whereas guilt may promote reparative behavior (Harder and Lewis 2013). Therefore, shame remains a distinct construct with predictive value even when other emotions are accounted for, justifying its independent investigation in our study.

Moreover, the experience of shame does not refer only to social exclusion due to inappropriate or socially unacceptable behavior (Lindsay‐Hartz 1984). Rather, it refers to situations in which a person evaluates themselves from the perspective of others and sees a valuable relationship at risk (Lewis 1986). Therefore, the individual may have obsessions with others’ judgments, which are likely replaced with effective communication (Asgarizade and Ghanbari 2022).

In addition, SP is related to self‐control (Mowlaie et al. 2016). Self‐control is a cognitive process that involves considering consequences and overcoming temptations (Tangney et al. 2004). Like other cognitive processes, self‐control is based in the frontal lobe and brain cortex. However, brain systems that work automatically and are based in the lower brain, such as the BIS, interfere with cognitive processes, including self‐control (Siegel 2007). Consequently, impulsive, hastily, and probably destructive behaviors due to a lack of self‐control influence an individual's life, including intimate relationships (Vohs et al. 2011; Kim et al. 2016). Additionally, shame and guilt have different relationships with self‐control and can be examined separately (Patrick et al. 2007).

Research has shown that self‐control has a significant effect on intimacy in couples (Haj Abootalebi et al. 2021) and is vital for decreasing aggression in romantic relationships (Gulledge et al. 2023). According to hot/cool system theory, two information processing systems (emotional and cognitive) are involved in the human brain (Lok et al. 2009). The emotional system, which is called the hot system, responds immediately after a stimulus is received, whereas the cognitive system, which is called the cool system, responds more slowly and is less severe (Metcalfe and Mischel 1999). On this basis, if the activity of the BIS is low, the SP would be lower, and consequently, self‐control would be more effective. When self‐control is increased, the cool system is more used, and behaviors are less impulsive and intense (Slessareva and Muraven 2004; Kim et al. 2016).

Previous research has suggested that both shame and self‐control may independently mediate the relationship between SP and interpersonal outcomes (McNaughton and Gray 2000). According to Gray's reinforcement sensitivity theory, SP is associated with heightened activity in the BIS, which increases individuals’ emotional sensitivity to potential threats that might cause shame and impair executive functioning, including self‐regulation (Baumeister et al. 2007). Although emotional processes, such as shame, and cognitive processes, such as self‐control, can influence one another (Baumeister et al. 2006), some findings suggest that they are triggered by distinct and parallel systems in response to punishment‐related cues (Tangney et al. 2004; Metcalfe and Mischel 1999; Mowlaie et al. 2016). Therefore, the present study conceptualizes shame and self‐control as parallel mediators, each reflecting a separate mechanism by which SP may affect intimacy.

Additionally, the brain pathways that influence shame and self‐control are often separate. Shame is an emotion experienced in the right hemisphere and in the subcortical area, whereas self‐control is a cognitive skill that occurs in the upper cortex of the brain. Therefore, SP has a different effect on each of these variables (Patrick et al. 2007; Siegel 2007).

Both self‐control and shame can be understood not only as relatively stable, trait‐like characteristics but also as dynamic, context‐dependent processes. Research indicates that self‐control may fluctuate depending on environmental demands and situational stressors (Baumeister et al. 2007; Na and Paternoster 2012), whereas shame is highly sensitive to interpersonal and contextual cues, emerging as a state emotion in response to perceived negative evaluations (Giovazolias and Paschalidi 2022; Asgarizadeh and Ghanbari 2022). This dual nature suggests that these mediators may not always operate simultaneously; rather, their influence on mental health may vary across different contexts and developmental periods. Considering this perspective provides a more dynamic framework for understanding the mechanisms underlying these pathways. Therefore, in this study, we examine the role of these two pathways with the awareness that they may operate in distinct temporal phases, which may help explain their complementary contributions.

This study examines the mediating role of shame and self‐control as two separate constructs, one emotion and the other cognitive variable, between SP and intimacy. Some studies have examined the relationships between SP and shame (Muris et al. 2015; Guimón et al. 2007), between SP and self‐control (Slessareva and Muraven 2004; Kim et al. 2016; Mowlaie et al. 2016), between shame and intimacy (Lutwak et al. 2003; Mitchell et al. 2008; Beck 2015), and between self‐control and intimacy (Vohs et al. 2011; Isanejad et al. 2016; Taralynne Brewer et al. 2018), but few studies have investigated these relationships via a mediation model. In addition, some studies have examined the mediation model with other emotions or self‐regulation (Gray and McNaughton 2000); however, no studies have concentrated on shame and intimacy in a mediation model. Although some studies have explained the relationship between shame and self‐control (Baumeister et al. 2007), they have not examined the associations with intimacy or SP.

Rewards and punishments are fundamental predictors of human behavior and emotional responses. Gray's biological and neurocognitive theory (1970) provides a framework for understanding how personality traits are shaped by rewarding and punishing stimuli. According to Gray's theory (1970), brain systems directly and indirectly influence behavioral patterns. Thus, it is essential to investigate these direct and indirect effects within the context of interpersonal relationships and to uncover the underlying mechanisms of this connection. By examining the functioning of the behavioral activation and inhibition systems in individuals, it becomes possible to predict personality tendencies and, through appropriate adjustment of interpersonal dynamics, mitigate the potential negative consequences that may arise in intimate relationships.

Intimacy, as a central component of interpersonal relationships, has been a focal point of interest for researchers, counselors, and therapists seeking to enhance individuals’ communication and relational functioning (Yoo et al. 2014). A lack of intimacy, however, can lead to significant challenges in personal life (Lutwak et al. 2003), many of which may stem from limited understanding of personality and behavioral processes. Although modest in scope, the present research aims to provide a general framework for examining behavior and its origins, thereby contributing to a better understanding of intimacy and its improvement.

This study aims to investigate the relationships between these variables in married individuals to identify factors that might affect intimacy and, consequently, to explore empowerment methods and behavior modification techniques for intimacy development and decreasing avoidance or fear of intimacy in the future. Owing to the importance of an individual's behavior in their relationships and the effects of these systems on many behaviors, it seems necessary to investigate the relationships between these brain systems and variables that might affect an individual's behavior. This helps us find a way to help individuals build better interpersonal relationships. The study hypotheses are as follows:

Hypotheses 1: SP has a significant effect on intimacy.
Hypotheses 2: Shame and self‐control can separately mediate the relationship between SP and intimacy.
Hypotheses 3: Shame and self‐control can mediate the relationship between SP and intimacy in a parallel mediation model.

## 2 Methods

### 2.1 Participants and Procedure

This research is performed via the correlation method. The population of this study was married people in Kerman. A total of 214 individuals were selected via the convenience sampling method. They were informed about the purpose of the study, and after providing informed consent from the sample, individuals who met the inclusion criteria, including literacy, being married, having spent at least 1 year living together, and living in Kerman city, responded to the research measures. Analysis was performed via Pearson's coefficient, a parallel mediation model, and a bootstrap test. Analysis was performed via SPSS23. The parallel mediation model was performed via process version 3 for SPSS.^1^

### 2.2 Measures

Sensitivity to Punishment and Sensitivity to Reward Questionnaire Revised and Clarified (SPSRQ‐RC): This questionnaire is designed to estimate (evaluate or examine) the brain systems mentioned in Gray's theory of personality. It consists of 20 items that are scored on a 5‐point Likert scale ranging from totally disagree (1) to totally agree (5). This questionnaire has two subscales: SP (odd items) and sensitivity to reward (SR) (even items). Higher scores in each part indicate greater sensitivity of the brain system. The first version of this questionnaire, included 48 yes/no items. In 2018, Conner et al. 2018 changed it to a shorter form judged on a 5‐point Likert scale. Cronbach's alphas for SP and reward were 0.86 and 0.80, respectively. In Iran, the psychometric properties were examined, and Cronbach's alphas for SP and SR were 0.83 and 0.70, respectively. In the current study, we examined only the SP subscale, and Cronbach's alpha for this subscale was 0.85.

Experience of shame scale (ESS): This questionnaire was designed to estimate different aspects of shame attitudes and traits and includes 25 items and three subscales: behavioral shame (9 items), characteristic shame (12 items), and bodily shame (4 items). The items are scored on a 4‐point Likert scale ranging from not at all (1) to very much (4). Cronbach's alphas for characteristic shame, behavioral shame, and bodily shame were 0.90, 0.87, and 0.86, respectively (Andrews et al. 2002). Additionally, there was good internal consistency (0.92) and test‐retest reliability (0.88). In this study, Cronbach's alpha values for the whole scale were 0.93 and 0.88 for characterological shame, 0.88 for behavioral shame, and 0.78 for bodily shame.

Tangney self‐control scale short form: This scale was designed with 35 items (Tangney et al. 2004). The short form used in this study contains 13 items scored on a 5‐point Likert scale ranging from never (1) to very much (5). Cronbach's alpha in this study is 0.83.

Personal assessment of intimate relationships questionnaire (PAIR): This scale was designed by Schaefer and Olson in 1981 and has six subscales for measuring different aspects of intimacy. Each subscale has six questions. This is scored on a 5‐point Likert scale ranging from not true in my relationship at all (1) to totally true in my relationship (5). Cronbach's alpha of the subscales has been reported to be above 0.70 (Schaefer and Olson 1981). In the present study, the total Cronbach's alpha coefficient was 0.87.

### 2.3 Data Analysis

The test was accomplished via SPSS v23. Pearson correlation analysis was used to study the correlation between the variables. Then, parallel mediation analysis was used (Hayes 2013) to examine the indirect effect of SP on intimacy through shame and self‐control. Indirect effects are considered significant if the confidence intervals do not contain zero. In addition, a 95% bias‐corrected confidence interval (95% BC CI) based on 5000 bootstrap samples was used to test the significance of the indirect effects.

## 3 Results

### 3.1 Preliminary Analysis

A total of 214 individuals participated in this research. Seventy‐five men (33.5%) and 139 women (62.8%) completed the questionnaire voluntarily. Fifty‐six individuals (24.7%) had diplomas, 93 (41.9%) had bachelor's degrees, 61 (26.5%) had master's degrees, and 4 (1.4%) had PhD. The age of the participants was between 20 and 66 years, and the average age of the subjects was 35.76 years. Additionally, participants between 1 and 44 years of age had passed since their marriage, and on average, 10.48 years had passed since their marriage.

The means and standard deviations (SDs) for each variable as well as their correlations are presented in Table 1. There is a negative and significant association between SP and intimacy. Shame has a positive and significant correlation with SP and a negative and significant correlation with intimacy. Self‐control has a negative and significant correlation with SP and a positive and significant correlation with intimacy. All correlations are significant; therefore, the mediation model can be performed.

**TABLE 1:** Descriptive statistics and correlation analysis of variables.

	*M*	SD	1	2	3	4
1. SP	28.84	7.98	—	0.57^**^	−0.26^**^	−0.21^**^
2. Shame	46.82	11.14	—	—	−0.39^**^	−0.28^**^
3. Self‐control	47.15	8.10		—	—	0.24^**^
4. Intimacy	125.08	25.22				—

Abbreviations: SD, standard deviation; SP, sensitivity to punishment.

^*^ *p* < 0.05.

^**^ *p* < 0.01.

### 3.2 Parallel Mediation Analysis

To assess the potential impact of common method bias, Harman's single‐factor test was performed. All the items from the main study variables were subjected to unrotated exploratory factor analysis. The results revealed that for the SPSRQ, the first factor accounted for 23.02% of the total variance; for the ESS, the first factor accounted for 30.26%; for the Tangney self‐control scale, the first factor accounted for 32.20%; and for the PAIR, the first factor accounted for 29.55%, which is below the commonly accepted threshold of 50% (Podsakoff et al. 2003). Therefore, common method bias is unlikely to have significantly affected the results of this study.

Age and marriage duration were examined as covariates. There was no significant effect for age (*R* ^2^ = −0.15, *p* = 0.24 > 0.05) or marriage duration (*R* ^2^ = 0.07, *p* = 0.60 > 0.05). Therefore, the parallel mediation model was analyzed with no covariates.

**FIGURE 1:**
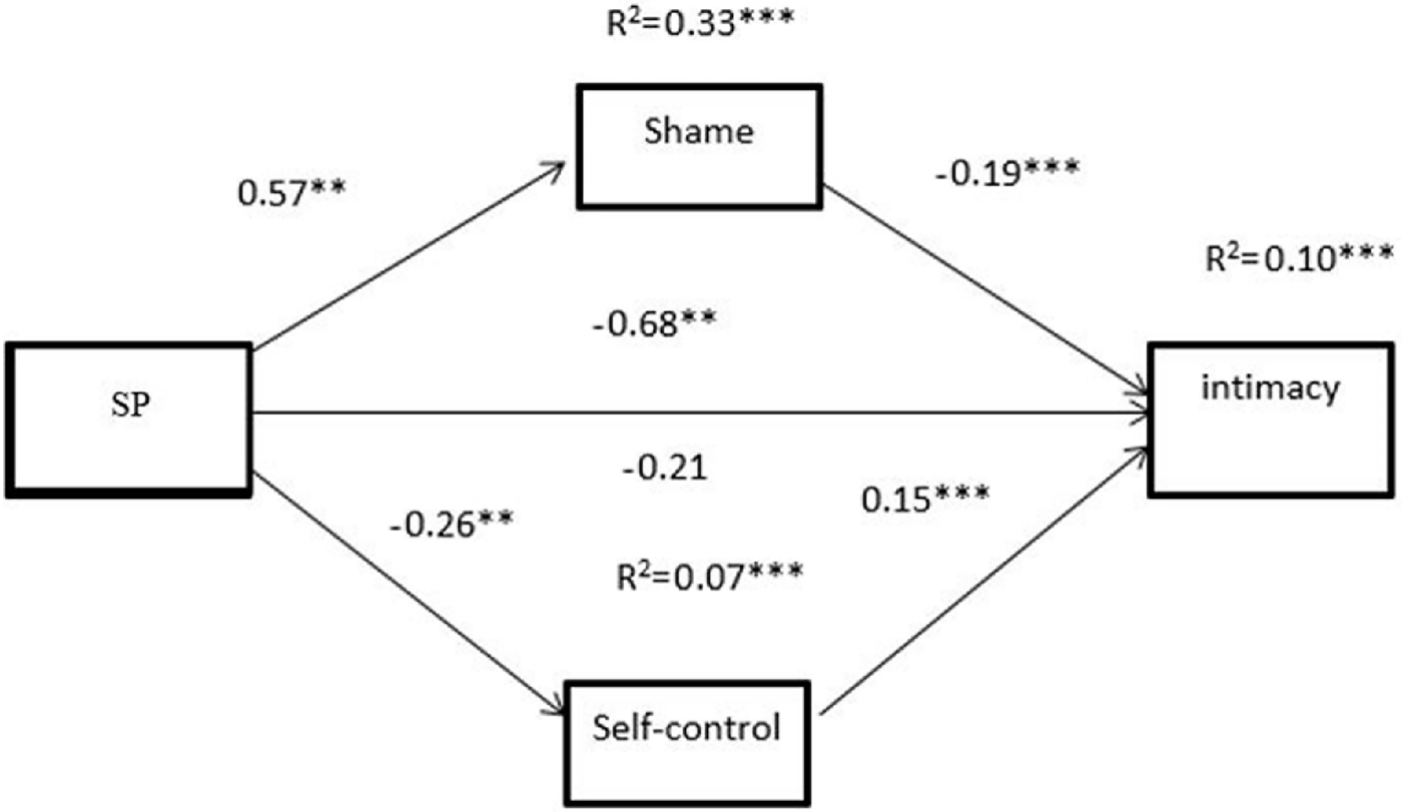
Parallel mediation model. SP, sensitivity to punishment. *p < 0.05; **p < 0.01; ***p < 0.001.

The total effect of SP on intimacy was significant (*b* = −0.68, SE = 0.21, *t* (212) = −3.19, *p* < 0.01). The total indirect effect (*b* = −0.47, SE = 0.14, 95% BC CI [−0.74, −0.21]) and the indirect effect of SP on intimacy through shame (*b* = −0.35, SE = 0.13, 95% BC CI [−0.61, −0.10]) and through self‐control (*b* = −0.12, SE = 0.07, 95% BC CI [−0.27, −0.01]) were significant. The direct effect of SP on intimacy was not significant (*b* = −0.21, SE = 0.25, 95% BC CI [−0.70, 29]). The results demonstrate that the total effect of SP on intimacy is significant. Although the direct effect was not significant, the indirect effect of SP on intimacy through shame and self‐control was significant. Therefore, sham control and self‐control can mediate this relationship. The means, SDs, and correlation matrices of the variables are presented in Table 2. All the results, including total effects, direct effects, and indirect effects, are illustrated in Figure 1.

**TABLE 2:** Mean (*M*), standard deviation (SD), and correlation matrix of the variables.

Dependent variable	*M*	SD	1	2	3	4
1. SP	28.91	7.93	—	0.57^**^	−0.26^**^	−0.21^**^
2. Shame	46.82	11.14		—	−0.39^**^	−0.29^**^
3. Self‐control	47.15	8.10			—	−0.24^**^
4. Intimacy	125.08	25.22				—

Abbreviation: SP, sensitivity to punishment.

^*^ *p* < 0.05.

^**^ *p* < 0.01.

## 4 Discussion

The purpose of this study was to examine the mediating role of shame and self‐control between SP and intimacy. The results demonstrated that the total effect of SP on intimacy and the indirect effects through shame and intimacy were significant, but the direct effect of SP on intimacy was not significant.

Consistent with other studies, this study demonstrated a significant association between SP and intimacy, and there was a significant effect through mediators (Shahzadi and Walker 2019; Meyer et al. 2005). However, the direct effect was not significant because there are several ways in which SP might affect intimacy, which can be explained in a “one‐way linear relationship.”

On the basis of Gray's reinforcement sensitivity theory (1970), when SP is high, an individual is more sensitive to unpleasant stimuli, pays more attention to them, and then forms defensive behaviors that might lead to obsession and compulsion, avoidance, increased anxiety, rumination, and worry (Corr 2008; Hundt et al. 2013). This sensitivity and consequent behaviors can affect intimacy in several ways. First, it might occur when a relationship begins. If an individual receives negative feedback or a person perceives rejection, he/she might completely avoid the same situations again because experiencing rejection repeatedly is intolerable and destroys his/her self‐esteem (Clark et al. 2015; Giovazolias and Paschalidi 2022). Second, after a relationship is started, a person with high SP does not feel safe. These individuals, who are very vulnerable to criticism, might feel anxious and frustrated due to small conflicts and experience many negative effects (Erdle and Rushton 2010). They might generalize this occasion and avoid expressing their needs and feelings on other occasions because they often expect negative feedback and bad things. This happens in different aspects, such as sexual and emotional aspects (Meyer et al. 2005; Asgarizadeh and Ghanbari 2022). Hence, high SP leads to negative affect, anxiety, and maladaptive behaviors. Additionally, mental vulnerability results in a decrease in self‐esteem and rumination and affects various aspects of life, including intimacy (Erdle and Rushton 2010; Hundt et al. 2013).

This research aimed to explain two important channels through which SP might affect intimacy. The first channel is through shame, which is an emotion that high SP might provoke (Muris et al. 2015). When this system receives negative or unpleasant stimuli and starts its activity, negative effects arouse and lead to different negative emotions, including shame. The relationship between shame and SP is positive and significant in this research, similar to previous studies (Guimón et al. 2007). Owing to an individual's vulnerability to punishment, rejection, and criticism, after receiving stimuli that cause such perceptions, shame is experienced immediately (Erdle and Rushton 2010). Shame comes to mind as others’ judgments, such as “I should be this way,” “If I behave this way, I'm bad,” or “This behavior caused embarrassment for me or others.” Sometimes, shame is adaptive and causes cognition and behavior improvement and an attempt to reach an ideal self (Lindsay‐Hartz 1984). However, if it increases abnormally, rather than being useful, it can cause harm, avoidance, fear of loss, and unloving (Lutwak et al. 2003; Muris et al. 2015; Asgarizadeh and Ghanbari 2022). Shame has a negative and significant correlation with intimacy, which is consistent with the findings of previous studies. An individual might feel shameful because he/she recedes from the ideal self and consequently has some problems in close relationships (Lindsay‐Hartz 1984; Fossum and Mason 1986; Beck 2015). If shame is more than normal, there will be feelings of being bad and having defects, and individuals will be anxious about being inadequate (Lutwak et al. 2003; Mckeogh et al. 2018). These thoughts and emotions keep individuals in a cycle. Shame prevents self‐disclosure. Hence, real feelings and needs are inhibited. This avoidance then receives negative feedback and results in intense shame and worthlessness (Mitchell et al. 2008).

There are issues that are less noticeable with respect to shame. Shame is an emotion that has long‐term mental and behavioral effects (Schore and Schore 2008). The closer a relationship is, the more information and knowledge are revealed about each other. Therefore, the experience of shame is more probable because the opinion and judgment of the person we are most interested in and associated with is very important to us and makes us more judgmental and less accepting or forgiving about ourselves (McKeogh et al. 2018; Giovazolias and Paschalidi 2022).

The second way evaluated in this study is the way information is processed. Sometimes, information processing is automatic, and the response is based on aroused emotions. The second processing way is controlled information processing, in which decisions are based on cognition. Gray's brain systems are completely responsive through affect (Metcalfe and Mischel 1999; Siegel 2007; Kim et al. 2016). As a result, increased self‐control might adjust the responses. Self‐control has a negative and significant correlation with SP (E. Slessareva and Muraven 2004; Kim et al. 2016). Self‐control can overcome temptation. It makes the individual consider different aspects and consequences and try and wait for a better outcome (Tangney et al. 2004). This can affect several factors in close relationships. An individual can control emotions and prevent severe conflicts and arguments (Vohs et al. 2011; Isanejad et al. 2016). It mentally stops rumination and negative thoughts, either. Additionally, it makes people attempt to solve a problem instead of avoiding it. In self‐control, a person is not ignored but expresses their feelings and needs adaptively (Haj Abootalebi et al. 2021) and might prevent aggression in romantic relationships (Gulledge et al. 2023). As relationships improve, assertiveness is reinforced (Taralynne Brewer et al. 2018). Notably, when the BIS is more sensitive, this does not mean that the individual does not have good information processing. They need more attempts and energy for emotion regulation and avoiding impulsivity and anxiety (Mowlaie et al. 2016).

An important consideration emerging from our findings is that the two mediation pathways may not act concurrently. Instead, it is plausible that they operate at different times, with each pathway becoming more influential under specific conditions or phases of the process. This temporal distinction could explain dynamic nature of shame (Giovazolias and Paschalidi 2022; Asgarizadeh and Ghanbari 2022) and self‐control (Baumeister et al. 2007; Na and Paternoster 2012) and the way they can mediate the relation between SP and intimacy. In addition, acknowledging shame and self‐control potential sequential or time‐dependent roles provides a more coherent interpretation of the results and aligns with broader theoretical perspectives on these variables.

This study was limited by the use of self‐report scales and personal questions and information that made the sampling process difficult and narrow. Additionally, the questions were not designed for Iran's culture and might have made some items vague for participants, for example, in defining sexual intimacy. The whole questionnaire was long and made the response less accurate. However, measuring these variables was inevitable.

## 5 Conclusion

The results of this study demonstrated that SP can affect intimacy through two different passes. One can arouse negative affect and emotions, such as shame, which can cause a sense of inadequacy in close relationships (Beck 2015). The other path is through information processing, which is automatic and sometimes harmful, but if self‐control increases, it can be better controlled (Izadpanah 2017). This study demonstrated separate paths for shame and self‐control, whereas some research has revealed a relationship between shame and self‐control (Baumeister et al. 2007). Future research should examine this relationship and clarify the present results.

## Author Contributions

**Zahra Mirzazade**: Data collection, data preparation and writing report **Ghlamreza Dehshiri**: Data analysis and study conceptualization. **Shaghayegh Zahraie**: Study conceptualization and writing report.

## Funding

The authors have nothing to report.

## Ethics Statement

This study involved no clinical intervention and was conducted using self‐report questionnaires. Participation was entirely voluntary, and informed consent was obtained from all 214 participants prior to their involvement. All responses were anonymous and could not be linked to identifiable individuals. This study was conducted in full accordance with the ethical principles and guidelines of the American Psychological Association (APA). The ethical standards were discussed and approved by the Department of Psychology at Al‐Zahra University.

## Conflicts of Interest

The authors declare no conflicts of interest.

## Data Availability

The data that support the findings of this study are available from the corresponding author upon reasonable request.

## Endnotes

1 The datasets are available from the corresponding author on reasonable request.
